# Pseudoprogression in microsatellite instability-high colorectal cancer during treatment with combination T cell mediated immunotherapy: a case report and literature review

**DOI:** 10.18632/oncotarget.18361

**Published:** 2017-06-03

**Authors:** Young Kwang Chae, Si Wang, Halla Nimeiri, Aparna Kalyan, Francis J. Giles

**Affiliations:** ^1^ Developmental Therapeutics Program of the Division of Hematology Oncology, Northwestern University, Chicago, Illinois, USA; ^2^ Feinberg School of Medicine, Northwestern University, Chicago, Illinois, USA; ^3^ Robert H. Lurie Comprehensive Cancer Center of Northwestern University, Chicago, Illinois, USA

**Keywords:** immune checkpoint inhibitors, metastatic colorectal cancer, immune-related response, anti-programmed death ligand-1 antibody, pseudoprogression

## Abstract

Evading tumor-mediated immunosuppression through antibodies to immune checkpoints has shown clinical benefit in patients with select solid tumors. There is a heterogeneity of responses in patients receiving immunotherapy, including pseudoprogression in which the tumor burden increases initially before decreasing to reach disease control. The characteristics and basis of pseudoprogression, however, remains poorly understood. We hereby report a case of microsatellite instability (MSI)-high metastatic colorectal cancer treated with combination of OX40 agonist and programmed death ligand-1 (PD-L1) antagonist that demonstrated pseudoprogression reaching 163% increase from baseline tumor burden. Tumor regression was subsequently observed and patient has remained in stable disease. Despite the substantial radiological progression, the symptomatic improvement reported by the patient led us to the decision of treatment continuation based on the suspicion of pseudoprogression, illustrating the importance of clinical evaluation in medical decision making while managing patients on immunotherapy. Additionally, the patient's MSI-high status contributes to his good, maintained response to PD-L1 blockade. Our case provides a frame of reference for fluctuation in tumor burden associated with pseudoprogression. Here we also evaluate the incidence and scale of pseudoprogression across solid tumor types.

## INTRODUCTION

Immunotherapy that restores antitumor response within the host is rising as a promising treatment option for cancer patients. As a part of the immune checkpoint system, the programmed death (PD) -1/PD ligand-1 (PD-L1) pathway is a critical mechanism which the tumor cells utilize to escape from immune destruction. Blockade of the PD-1/PD-L1 pathway through antibodies to PD-1 has demonstrated encouraging potential in treating advanced solid tumors [1]. During treatments of immune checkpoint inhibitors such as antibodies against PD-1 or cytotoxic T lymphocyte-associated antigen-4 (CTLA-4), tumor responses were observed and assessed using Response Evaluation Criteria in Solid Tumors (RECIST) [2]. Some patients, however, experience immune-related responses such as initial tumor growth or appearance of new lesions followed by reduction in tumor burden. For example, in a subgroup of melanoma patients treated with ipilimumab, an anti-CTLA-4 monoclonal antibody, lesion enlargement preceded a decrease in tumor burden. The biopsy of the enlarged lesions revealed inflammatory cell infiltrates or necrosis [3]. This pattern of delayed clinical responses, or pseudoprogression, is recognized by clinicians and researchers as unconventional and characterized by tumor regression occurring or being maintained in the presence of new lesions or after initial radiological evidence of progressive disease (PD) determined by RECIST [4–6].

Such findings of pseudoprogression are not unique to immune-targeted treatments. They have been first described with tyrosine kinase inhibitors such as imatinib that result in tumor enlargement with decreased tumor density. Size-based RECIST is inadequate in recognizing change in tumor density as a parameter for treatment response, hence prompting the development of Choi criteria [7]. Similarly, response to bevacizumab, an antiangiogenic agent, may not be adequately assessed by RECIST because changes in tumor morphology on computed tomography (CT) were shown to be more significantly related to overall survival than tumor size [8]. With immunotherapeutics on the rise, pseudoprogression poses a challenge to clinicians who wish to accurately evaluate the clinical efficacy of these novel agents. The frequency, depth, developmental patterns, and predisposing factors of pseudoprogression still remain largely unknown to date. To further characterize pseudoprogression, increased reporting of this unconventional pattern of response in ongoing trials with immunotherapeutics will be beneficial.

We present the case of a 61-year-old male with metastatic adenocarcinoma of the colon treated with OX40 agonist and PD-L1 antagonist, who experienced initial increase in size and number of tumor lesions with subsequent tumor regression. We will also provide a brief literature review of the frequency and scale of pseudoprogression during treatment with FDA-approved and additional immune checkpoint inhibitors.

## CASE PRESENTATION

The patient first presented with microcytic anemia and guaiac-positive stool during routine physician visit. Colonoscopy revealed a proximal lesion of the cecum that was biopsied and positive for adenocarcinoma. Two months later he underwent a laparoscopic assisted right hemicolectomy, with surgical pathology revealing well-differentiated, low-grade adenocarcinoma with no lymph node involvement (staged T2N0). There were also histologic features suggestive of MSI including mild to moderate intratumoral lymphocytic response, moderate peritumor lymphocytic response and <5% mucinous tumor component. In follow-up CT local recurrence was noted at the anastomotic site as well as new perirenal nodules. Four months after surgery, the patient complained of abdominal pain with radiation to the back. He then underwent transverse/descending colon cancer resection with biopsy demonstrating poorly-differentiated, high-grade adenocarcinoma with lymph-vascular invasion (staged T3N1b). Two of 19 lymph nodes were found to be positive for disease. Immunohistochemistry of the tumor specimen demonstrated loss of MLH1 and PMS2, and retention of MSH2 and MSH6. Further analysis revealed negative MLH1 hypermethylation but heterozygous mutation in KRAS codon 13. Subsequent imaging revealed peri-nephric mass, hepatic lesions and mesenteric lymph node involvement. The patient later underwent comprehensive genomic profiling with next generation sequencing (Table 1).

**Table 1:** Mutations detected in the patient and their functional implication status

Disease relevant gene	Alteration identified	Possible functional implication ^a^
KRAS	G13D	Yes
TET2	K95fs*18, N439fs*4	Yes
BRCA2	K1691fs*15	Yes
CEBPA	H24fs*84	Yes
CTNNB1	S45F	Yes
FBXW7	L234fs*5	Yes
TP53	P222L	Yes
ARID1A	P224fs*8	Yes
ASXL1	G645fs*12	Yes
CDH1	A634fs*29	Yes
MLH1	V612fs*2	Yes
NOTCH2	S1419fs*8	Yes
ALK	E310D	No
ARID2	R1679Q	No
ATR	P315T	No
BARD1	P358_S364del	No
CTNNA1	R540H	No
ESR1	R269C	No
FGFR3	K403fs*93	No
IL7R	I121fs*1	No
IRF4	K302E	No
IRS2	N21del, P1225L	No
JAK1	P861fs*4	No
JAK2	V984M	No
KDM6A	R621C	No
MLL2	P565L	No
MYC	G301D	No
MYCN	P237L	No
NF1	R1870W	No
NOTCH1	H196R	No
NTRK3	M452V	No
PIK3CG	L91M	No
PIK3R1	I292N, K593E	No
PTCH1	E44del	No
RB1	R910Q	No
RUNX1T1	R336H	No
SETD2	L2486M	No
SPEN	I2469V, P255del	No
TGFBR2	T230M	No

^a^ Functional implication status was reported as part of the patient's genomic profile from Foundation Medicine.

The patient underwent 7 cycles of folinic acid with fluorouracil and oxaliplatin plus bevacizumab following surgery and discontinued due to neuropathy. Post-chemotherapy CT scan revealed 50% shrinkage of liver lesion and persistence of peri-nephric mass. He was subsequently transitioned to folinic acid with fluorouracil and irinotecan plus bevacizumab for one month. A right radical nephrectomy was performed two months later to remove the right retroperitoneal mass and resect the small bowel and omental flap. Surgical pathology returned with grade III infiltrating colonic adenocarcinoma with areas of necrosis in the retroperitoneal mass of the right kidney (8.0 × 7.2 × 5.2 cm) and a firm red mucosal segment of the small bowel (3.8 × 3.0 cm) adherent to the mass. New baseline CT scan two months after operation revealed no evidence of disease in chest, abdomen or pelvis. The patient was subsequently initiated on adjuvant chemotherapy of capecitabine plus oxaliplatin and developed increasing sciatica pain after 3 cycles. CT revealed recurrence in right psoas muscle and possible liver lesion metastases. He then underwent palliative radiotherapy for right psoas mass followed by 6 cycles of folinic acid with fluorouracil and irinotecan plus aflibercept, but experienced intolerance with diarrhea and fatigue. His carcinoembryonic antigen level was within normal limit at the beginning of this last round of chemotherapy (2.7 ng/mL).

**Figure 1:**
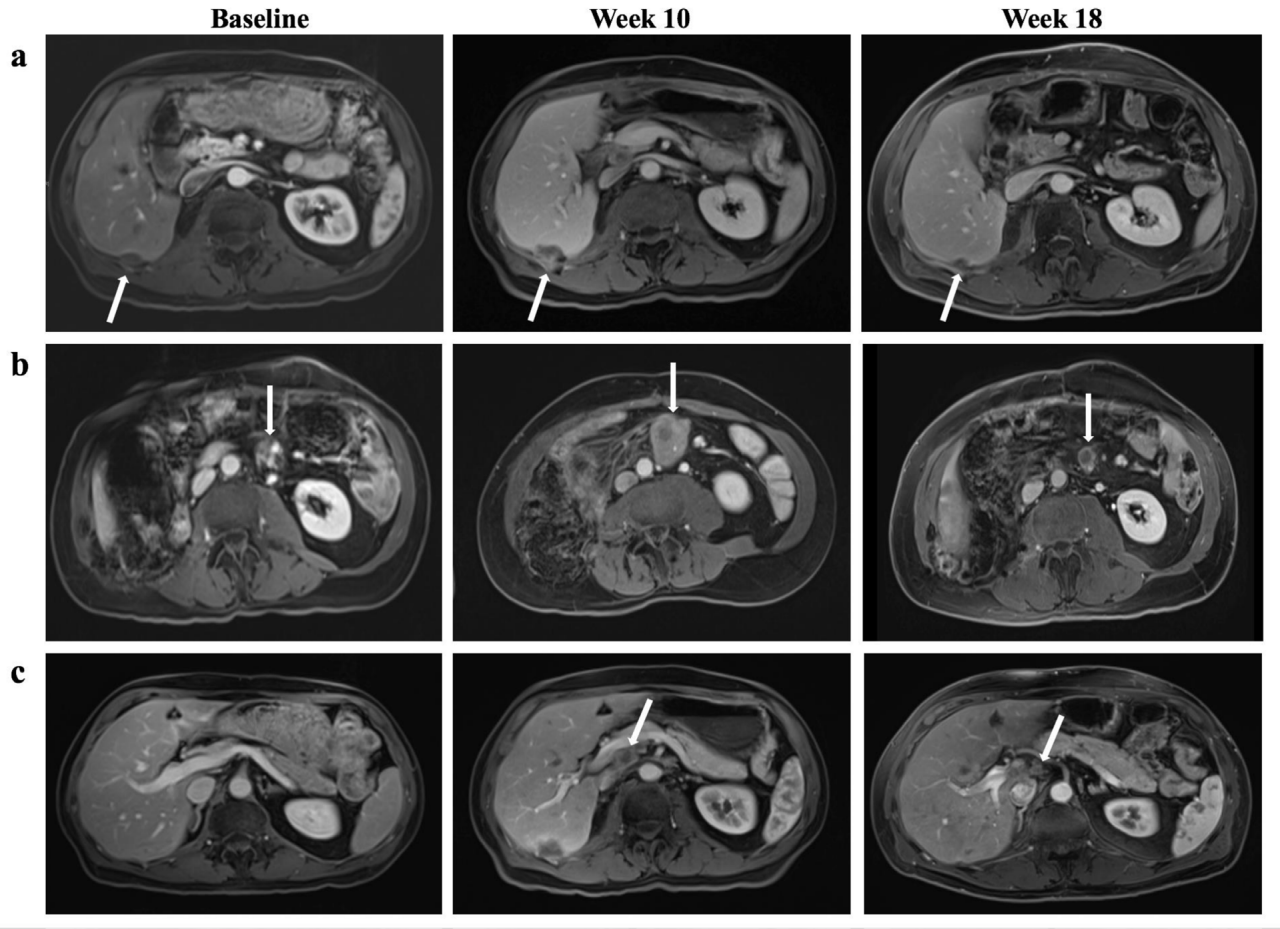
Tumor response to combined OX-40 agonist and PD-L1 antagonist regimen **a.** The right hepatic lobe surface metastasis (white arrow) was significantly increased in size at week 10, measuring 2.2 × 2.4 cm compared with 1.8 × 1.0 cm on baseline. At week 18, the lesion shrunk to 1.7 × 1.2 cm. **b.** The mesenteric metastasis (white arrow) grew from 2.0 × 1.4 cm at baseline to 4.3 × 3.7 cm at week 10, and subsequently decreased to 1.4 × 2.1 cm at week 18. **c.** A new periportal lymph node (white arrow) was detected at week 10 that measured to be 2.2 × 1.7 cm and subsequently became 0.8 × 0.9 cm with central necrosis at week 18.

**Figure 2:**
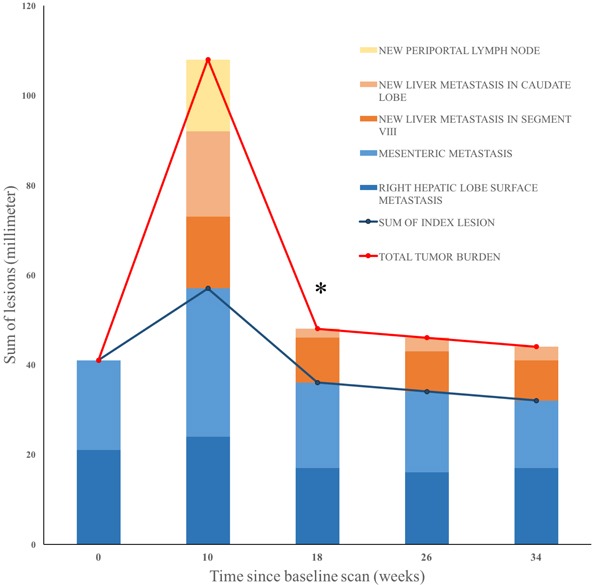
Change in tumor burden under combined treatment of OX40 agonist and PD-L1 antagonist over time The diameters of new and existing lesions were measured in millimeters every eight to ten weeks. Blue blocks denote baseline lesions. Orange blocks are new metastatic lesions, and yellow are new lymphadenopathy. * The periportal lymph node detected at week ten was not included in overall tumor burden at week 18 because it decreased to less than 10mm.

The patient was enrolled in a clinical trial in year 2 with combination treatment of OX40 agonist and PD-L1 antagonist administered every two weeks. Major findings from baseline magnetic resonance imaging (MRI) scan included right hepatic lobe surface metastasis (1.8 × 1.0 cm) and mesenteric metastasis (2.0 × 1.4 cm) (Figure 1a and 1b). Lactate dehydrogenase (LDH) level was normal prior to treatment (201 unit/L) and raised slowly once treatment started. LDH level was abnormally high at week seven and nine (242 unit/L and 258 unit/L respectively). Follow-up MRI at week ten demonstrated enlarged right hepatic lobe surface metastasis (2.2 × 2.4 cm) and mesenteric metastasis (4.3 × 3.7 cm), along with numerous new liver metastases and new periportal lymphadenopathy (2.2 × 1.7 cm) (Figure 1). One of the new metastases in the caudate lobe measured to be 2.0 × 1.9 cm (Figure 2). An increase of 163% from baseline tumor burden was observed (Figure 2). Despite the radiological progression, the patient was doing well with no new complaints. Intriguingly he reported improvement in abdominal and back pain with most lab parameters being reasonably within limits. After discussion with the patient, the decision was made to continue on current treatment in suspicion of pseudoprogression. In following weeks, LDH level dropped to be within normal limits (ranging from 144 - 181 unit/L). Subsequent MRI at week 18 showed an excellent response with shrunken right hepatic lobe surface metastasis (1.7 × 1.2 cm) and mesenteric metastasis (1.4 × 2.1 cm) (Figure 1a and 1b). The previously seen periportal lymph node was resolved with central necrosis (0.8 × 0.9 cm) (Figure 1c). The caudate lobe mass decreased to 0.2 × 0.3 cm (Figure 2). MRI at week 34 continued to reveal slight decrease of tumor burden and the patient continued to report feeling well to date (liver metastasis 1.7 × 0.7 cm, mesenteric metastasis 1.5 × 1.2 cm, lymph node undetectable) (Figure 2).

**Table 2:** Unconventional response rates and magnitude of increase in tumor burden from baseline for CTLA-4, PD-1 and PD-L1 inhibitors across solid tumors

Agent	Mechanism of action	Trial	Cancer Type	No. of Evaluable Patients	No. of unconventional Responses	Unconventional Response Rate (%)	Maximum increase from baseline tumor burden (%) ^a^	Primary Tumor response criteria
Ipilimumab	Anti-CTLA-4 monoclonal antibody	Wolchok *et al* (2009) [4]	Melanoma	227	22	9.7	113	irRC
		O’Day *et al* (2010) [16]	Melanoma	155	12	7.7	Not reported	irRC
Tremelimumab	Anti-CTLA-4 monoclonal antibody	Ribas *et al* (2012) [17]	Melanoma	36	1	2.8	Not reported	RECIST 1.0
		Calabro *et al* (2015) [18]	Mesothelioma	29	2	6.9	Not reported	irRC
Pembrolizumab	Anti-PD-1 monoclonal antibody	Ribas *et al* (2015) [19]	Melanoma	360	57	15.8	Not reported	RECIST 1.1
		Hodi *et al* (2016) [13]	Melanoma	327	24	7.3	80	RECIST 1.1
		Seiwert *et al* (2016) [20]	Head and neck squamous cell carcinoma	56	1	1.8	Not reported	RECIST 1.1
Nivolumab	Anti-PD-1 monoclonal antibody	Hodi *et al* (2014) [6]	Melanoma	107	4	3.7	Not reported	RECIST 1.0
		Robert *et al* (2015) [21]	Melanoma	206	17	8.3	63	RECIST 1.1
		Weber *et al* (2015) [5]	Melanoma	120	10	8.3	25	RECIST 1.1
		Gettinger *et al* (2016)[9]	Non-small-cell lung cancer	52	3	5.8	20	RECIST 1.1
Atezolizumab	Anti-PD-L1 monoclonal antibody	Powles *et al* (2014) [22]	Urothelial carcinoma	67	1	1.5	10 ^b^	RECIST 1.1
		McDermott *et al* (2016) [23]	Renal cell carcinoma	70	4	5.7	80	irRC and RECIST 1.1
		Rosenberg *et al* (2016) [24]	Urothelial carcinoma	310	20	6.5	95	irRC and RECIST 1.1
Durvalumab	Anti-PD-L1 monoclonal antibody	Massard *et al* (2016) [25]	Urothelial carcinoma	42	3	7.1	75	RECIST 1.1
Avelumab	Anti-PD-L1 monoclonal antibody	Kaufman *et al* (2016)[26]	Merkel cell carcinoma	65	1	1.5	60	RECIST 1.1

Unconventional response is defined as tumor regression occurring or being maintained in the presence of new lesions or after initial radiological evidence of tumor growth.

^a^ Changes in tumor burden were estimated from published spider plots. The published graphs, from which the percent increase in this column was estimated, were based on unidimensional tumor measurements, except the ones in Wolchok et al [4].

^b^ This patient who initially responded to treatment later presented with new lesions that were consistent with pseudoprogression with a biopsy of extensive necrosis. The maximum increase in tumor burden was estimated from the tumor assessment prior to appearance of new lesions instead of from baseline.

## DISCUSSION

To our knowledge, a scale of pseudoprogression that reached 163% increase from baseline as demonstrated in our case has not been reported previously. Our patient was found to have substantial radiological progression soon after he was administered a combination of OX40 agonist and PD-L1 inhibitor. Appearance of new lesions and enlargement of target lesions, leading to 163% increase from baseline in overall tumor burden, would have been characterized as disease progression according to RECIST (Figure 2). Per the immune-related response criteria (irRC), this patient would have also been characterized as disease progression (irPD), but confirmation requires a repeat scan within four weeks. The patient, however, had improving clinical presentation that mismatched with the radiological evidence of disease. The disconnection between clinical and radiological presentations thus raised the suspicion of pseudoprogression. Subsequent scans demonstrated a steady trend of decline in tumor burden, confirming previous findings as pseudoprogression and rejecting the previous assessment of irPD. The patient has remained in stable disease to date per irRC.

This case is unique for two reasons. First, it shows extraordinary scale of pseudoprogression, with 163% of tumor growth from baseline. In addition to the substantial enlargement of target lesions, the size of new liver metastases when they first appeared was also notable. The mass in the caudate lobe initially presented as a considerable lesion with its dimensions being 2.0 × 1.9 cm, contributing considerably to the increase in overall tumor burden at the time. This is unusual for new lesions because they typically first appear small before growing into larger ones. The magnitude of maximum increase in tumor burden associated with pseudoprogression reported previously ranges from 20% - 113% (Table 2) [4, 9]. Our case contributes to further elucidating pseudoprogression by serving as a reference point for the extent of tumor growth. Second, even with substantial increase in tumor burden including the emergence of new large lesions, the symptomatic improvement experienced by the patient is a key reason why we continued the treatment regimen. Recently hyperprogressive disease was observed in a small subset of patients treated with anti-PD-1/PD-L1 monotherapy, which is associated with older patients (> 65 years old) and slower growing tumors at baseline [10]. It is difficult to differentiate pseudoprogression from hyperprogression based on radiological evidence alone when they first emerge. Treating physicians therefore need to rely on other aspects of the clinical data to evaluate tumor response. In our case, the patient's pain almost disappeared and fatigue resolved with increased overall level of energy. Had the dramatic radiological change been real disease progression or hyperprogression, the patient's condition would have probably deteriorated instead. This demonstrates that in addition to radiologic criteria, clinical evaluation is also a critical part of medical decision making regarding patients on immune checkpoint inhibitors.

Not only is the scale of “tumor flare” significant in this case, but the degree of tumor shrinkage following the flare within eight weeks is also worth noting. One of the new lesions, a periportal lymph node, measured to be 2.2 × 1.7 cm at week ten after treatment but subsequently shrunk to 0.8 × 0.9 cm with central necrosis evident on MRI at week 18 (Figure 1c and Figure 2). The lymph node was so small that it was not included in future calculation of tumor burden. Similarly, one of the target lesions at baseline, the mesenteric metastasis, doubled both of its dimensions at the first scan post-treatment and decreased to a size smaller than baseline eight weeks later (Figure 1b). The relatively fast and major reduction in tumor size provides a frame of reference regarding how much fluctuation in tumor burden can be expected in pseudoprogression.

Change in LDH level reasonably correlates with the change in overall tumor burden in this case. A rise of LDH level to being abnormally high was observed around the same time when pseudoprogression was detected. The decline of LDH level back to normal range also paralleled the shrinkage of tumor time wise. It is not clear at this point how to interpret the relationship of LDH level and radiological progression observed in this case, but it may be clinically relevant to study the role of LDH in differentiating pseudoprogression and real progression in the setting of immune-targeted treatment.

Another notable feature of our patient is that his MSI status is high, which may explain his excellent response to treatment of immune checkpoint inhibitors compared to chemotherapy. His tumor specimen demonstrated loss of MLH1 and PMS2 and retention of MSH2 and MSH6, indicating loss of normal DNA mismatch repair function within the tumor. The patient also had a lot of somatic mutations evident from the genetic profiling report (Table 1). All information points to very high MSI status and indicates the deficiency of tumors at mismatch repair. Le *et al.* found that the mismatch repair-deficit tumors respond better to PD-1 blockade than mismatch repair-proficient tumors regardless of tumor type [11]. This may be explained by the fact that the mismatch repair-deficient tumors create a microenvironment full of immune checkpoint ligands including PD-1, PD-L1 and CTLA-4 to evade tumor elimination [12]. Given the high MSI status of our patient, it is not surprising to see him responding well to a treatment involving PD-L1 antagonist. It is worth to investigate, however, whether having MSI-high status would likely cause more pseudoprogression as compared to MSI-stable cancer.

Pseudoprogression with immune checkpoint inhibitors has been observed in previous reports and would have been misclassified as progressive disease according to size-based WHO or RECIST criteria, therefore prompting the development of irRC [4]. Applying irRC, it was found that 9.7% of melanoma patients (22 of 227 patients) treated with ipilimumab demonstrated pseudoprogression that would have been prematurely classified as PD by WHO criteria [4]. Similarly, 7.3% of patients (24 of 327) who received pembrolizumab (anti-PD-1 antibody) for treatment of advanced melanoma experienced early or delayed pseudoprogression, including tumor regression and stable disease despite new lesion development, as well as temporary increase in the size of target lesions [13]. One study reported that 3.7% of metastatic melanoma patients (4 of 107 patients) treated with nivolumab (anti-PD-1 antibody) had unconventional response patterns suggestive of pseudoprogression [6]. During treatments of solid tumors with antibodies against CTLA-4, PD-1 or PD-L1, mean incidence of unconventional immune-related responses, or pseudoprogression is 6.3% (range 1.5 - 15.8%) (Table 2). PD-1 inhibitors appear to have a higher rate of pseudoprogression (mean 7.3%, range 1.8 - 15.8%) than anti-CTLA-4 and anti-PD-L1 agents (mean 6.8% and 4.5%, range 2.8 - 9.7% and 1.5 - 7.1% respectively). In addition, although the report of the degree of pseudoprogression in combined immunotherapy is limited, it was observed that 7.7% of patients (4 of 52 patients) with advanced melanoma receiving nivolumab plus ipilimumab had immune-related partial response or stable disease [14]. Our case report contributes to further delineating the frequency of such atypical response pattern in dual immunotherapy, but more clinical data is needed to fully understand this phenomenon. Regardless of whether immunotherapeutic agents are used alone or in combination, it is evident that solely relying on conventional criteria such as RECIST runs into the risk of inadequately evaluating the tumor response to immune checkpoint inhibitors for a small subgroup of patients.

Although pseudoprogression is becoming increasingly recognized by clinicians and researchers, the basis of this phenomenon is still not fully understood. Evidence such as biopsy that revealed inflammatory cell infiltrates and necrosis in the enlarged lesions supports the idea that pseudoprogression may be “tumor inflammation” instead and clinical response may be delayed [3, 15]. Because current imaging technique cannot differentiate pseudoprogression from real disease progression, clinicians should take extra caution when evaluating each patient's response to immunotherapeutic agents. The goal is to avoid either premature withdrawal of the treatment or prolonging ineffective treatment. Due to a paucity of information on pseudoprogression, currently there is no means to predict whether a patient will demonstrate pseudoprogression when treated with immune checkpoint inhibitors. Further studies are needed to help delineate the characteristics of patients who experienced pseudoprogression. With this information, clinicians will be more prepared when managing patients with a high possibility of encountering pseudoprogression on immunotherapy. It remains unknown whether patients who experienced pseudoprogression remain in disease control longer than those who did not. A meta-analysis regarding the long term response is ongoing by our research team currently.

As immune checkpoint inhibitors, especially anti-PD-1 and anti-PD-L1 agents, become more widely used by clinicians, the task of appropriately evaluating tumor response remains a challenge in patient management. Multiple studies using antibodies against CTLA-4, PD-1 and PD-L1 provide the basis of evaluating the incidence and scale of pseudoprogression across a variety of solid tumors [4–6, 9, 13, 16–26] (Table 2). Our case, however, is the first to demonstrate pseudoprogression during combinational treatment of PD-L1 inhibitor and OX40 agonist in MSI-high metastatic colorectal adenocarcinoma. It also represents a much larger tumor burden increase (163% from the baseline) than what has been previously published (113%) [4]. Further research is needed to delineate the basis of pseudoprogression in the usage of immune checkpoint inhibitors, differentiate pseudoprogression from real progression, as well as characterizing predisposing factors of pseudoprogression.
